# Ocular surface characteristics and its association with soft contact lens fitting

**DOI:** 10.1111/opo.13559

**Published:** 2025-08-27

**Authors:** Javier Rojas Viñuela, James S. Wolffsohn, Alejandra Consejo, Eef van der Worp, David P. Piñero

**Affiliations:** ^1^ Natural Optics Balaguer Lleida Spain; ^2^ School of Optometry Aston University Birmingham UK; ^3^ Aragon Institute for Engineering Research University of Zaragoza Zaragoza Spain; ^4^ Eye‐Contact‐Lens Research and Education Amsterdam the Netherlands; ^5^ Department of Optics, Pharmacology, and Anatomy University of Alicante Alicante Spain; ^6^ Department of Ophthalmology Vithas Medimar International Hospital Alicante Spain

**Keywords:** contact lens movement, corneo‐scleral topography, sagittal height, silicone hydrogel contact lens, soft contact lens

## Abstract

**Purpose:**

To analyse the associations between ocular surface shape parameters and soft contact lens fitting.

**Methods:**

A total of 106 eyes of 53 participants (53 right and 53 left eyes) fitted with standard silicone hydrogel soft lenses were analysed retrospectively. The sagittal height of the lenses was obtained from an independent publication. The lens fit was analysed objectively with proprietary software and corneo‐scleral parameters were obtained with the Eye Surface Profiler. The relationship between the sagittal height of the eye (OC‐SAG) and the lens (CL‐SAG) was defined as the delta‐sag (δ‐sag) and characterised for this group.

**Results:**

The OC‐SAG and δ‐sag were in the range of 3600 and 400 μm, respectively, with no statistically significant differences between the right and left eyes. There were statistically significant differences between the corneoscleral junction (CSJ) angle of the right and left eyes (*p* = 0.002). The nasal portion showed the sharpest transition in both eyes and was significantly different between the right and left eyes only in that specific quadrant (*p* < 0.001). The peripheral cornea (α) was steeper with a mean value around 38°. The proximal sclera (β) showed a flatter slope close to 36° in both eyes. Following the pattern of the CSJ angle, there were statistically significant differences only between the nasal‐α of the right and left eyes (*p* < 0.001). There were no significant correlations between the δ‐sag and the lens fitting features in either eye (*p* > 0.05). Some moderate and strong associations between the lens fit and peripheral ocular parameters were found, although they were not consistent between the right and left eyes.

**Conclusion:**

Soft contact lens movement seems to be influenced by the corneo‐scleral transition at the superior and inferior quadrants. It would be better to focus on the slopes in these peripheral areas rather than the central corneal parameters or the sagittal height.

**Key points**

- Post‐blink soft contact lens movement in primary gaze and push‐up recovery speed seem to be influenced by the corneo‐scleral transition at the superior and inferior quadrants.
- Peripheral corneo‐scleral parameters influenced the soft lens fit, but there was no association with central corneal parameters.
- Some patterns of soft lens fitting related to corneo‐scleral parameters can be described, but they were not consistent between the right and left eyes. This could be attributed to the complexity of the on‐eye soft lens performance.

## INTRODUCTION

Contact lens discomfort (CLD) is a complex and multifactorial issue that has been studied extensively.^1^ Suboptimal soft contact lens fitting has been identified as one factor involved in CLD.^1^, ^2^ Specifically, participants with loose fitting or more mobile lenses report reduced comfort.^3^, ^4^

Currently, there is no established way to predict on‐eye soft lens performance, and the number of factors involved makes it a challenging task. It is well known that selection of the lens base curve, based on central keratometry, does not correlate well with lens fit.^5^, ^6^ The use of peripheral corneal data from topography or videokeratoscopy may improve the prediction, but a trial fitting is still preferred to select the initial lens.^5^ The latest evidence suggests that extending measurement of the eye surface profile to include the corneoscleral junction (CSJ) profile using anterior segment optical coherence tomography (AS‐OCT) accounts for more of the variance in soft lens fitting than corneal topography alone. However, most of the variance in the soft lens fitting is still unclear.^7^

Recently, a trend of fitting soft lenses using the sagittal depth of the lens (CL‐SAG) and the sagittal height of the eye (OC‐SAG) has re‐emerged.^8^ The delta‐sag (δ‐sag) parameter is defined as the difference between CL‐SAG and OC‐SAG.^8^ However, it is unclear what the optimum δ‐sag ranges are for an acceptable fit, or how the on‐eye lens performance varies when this parameter is altered.^9^

Although AS‐OCT was instrumental in providing the earliest objective findings of the CSJ profile, this technology provides information on just one meridian at a time. Therefore, multiple measurements are needed to obtain information on different meridians, and they are assessed manually with a calliper, manipulated by the examiner.^9^ More recently, scleral topographers or profilometers have become available, allowing automatic measurement of scleral asymmetry in 360°, and are now preferred to characterise the CSJ profile.^9^ Fully objective and automated algorithms to demarcate the limbus position and characterise the CSJ transition in 360° have been developed for some of these profilometers.^10^, ^11^ Additionally, there is some evidence that the measured mean values of OC‐SAG obtained using profilometry differ both statistically and clinically from those calculated from the corneal parameters.^12^

With regard to soft lens fitting on the peripheral ocular surface^5^, ^6^, ^7^ and the availability of new devices to map that area automatically, the objectives of the current study were: (1) to describe the ocular surface shape in a group of existing soft contact lens wearers using a commercially available profilometer and (2) to analyse the associations between ocular shape parameters and soft contact lens fitting, in order to determine whether on‐eye soft lens behaviour is related to the ocular surface profile. CL‐SAG data obtained from an independent publication^13^ were used to describe the δ‐sag pattern. Objective assessment of CL centration, movement and push‐up recovery were used to analyse how varying δ‐sag values may impact on‐eye lens performance and to look for correlations with any of the corneo‐scleral parameters.

## METHODS

### Participants

A total of 106 eyes from 53 healthy Caucasian participants (53 right and 53 left eyes) fitted with standard silicone hydrogel soft contact lenses from a single centre were analysed retrospectively. Participants comprised 17 males (32%) and 36 females (68%), aged 18–59 years (mean age 29 ± 11 years). Participants wearing standard bi‐weekly or monthly reusable silicone hydrogel lenses in both eyes as their main form of vision correction and who regularly attended follow‐up visits were included in the study. All participants had worn the lenses for a minimum of 1 hour at the time of the evaluation. Participants wearing spherical, toric or multifocal standard soft lenses to correct any amount of myopia, hyperopia, astigmatism or presbyopia were included. The mean ± SD distance contact lens prescription at the time of the examination was −1.88 ± 2.75 D sphere, −0.88 ± 0.28 D cylinder at 107.0° ± 70.8° in the right eye and −1.64 ± 2.93 D sphere, −1.15 ± 0.46 cylinder at 129° ± 59° in the left eye. Participants with previous corneal surgery, corneal ectasia or any form of corneal irregularity, who were suffering from a systemic condition that could affect the physiology of the eye, who were being treated with a medication that could affect the ocular tissues and/or who were wearing rigid gas permeable contact lenses were also excluded from the study.

The study methods adhered to the tenets of the Declaration of Helsinki and were approved by the ethics committee for medical research of the Health Department of Alicante (General Hospital, Alicante, Spain; CEIm 2021‐105, ISABIAL 2021‐0224).

### Contact lenses

In total, 51% of the eyes (54 eyes) wore standard spherical soft lenses, 40% (42 eyes) wore toric lenses and 9% (10 eyes) wore multifocal lenses. The lenses worn successfully by the participants at the time of the follow‐up visit were: Biofinity, Biofinity Toric, Biofinity Multifocal (Coopervision, coopervision.com), PureVision 2 HD, PureVision 2 HD for Astigmatism, PureVision 2 HD for Presbyopia, Ultra, Ultra for Astigmatism, Ultra for Presbyopia (Basuch+Lomb, bausch.com), Air Optix, Air Optix for Astigmatism (Alcon‐alcon.com), Oasys and Oasys for Astigmatism (Johnson & Johnson, jnj.com). The posterior CL‐SAG for each of these lenses was obtained from an independent publication. These measurements had been taken at room temperature^13^ (Table 1).

**TABLE 1:** Sagittal height of the contact lenses (CL‐SAG) included in the study.

Lens	Eyes (%)	CL‐SAG (μm)	Diameter (mm)	Base curve (mm)	Material
Biofinity (CooperVision)	24 (23%)	3688	14.0	8.60	Comfilcon A
Biofinity toric (CooperVision)	29 (27%)	3864	14.5	8.70	Comfilcon A
Biofinity multifocal (CooperVision)	4 (4%)	3707	14.0	8.60	Comfilcon A
Ultra (Bausch + Lomb)	10 (9%)	3812	14.2	8.50	Samfilcon A
Ultra for astigmatism (Bausch + Lomb)	2 (2%)	3969	14.5	8.60	Samfilcon A
Ultra for presbyopia (Bausch + Lomb)	4 (4%)	3812	14.2	8.50	Samfilcon A
PureVision 2 HD (Bausch + Lomb)	2 (2%)	3627	14.0	8.60	Balafilcon A
PureVision 2 HD for astigmatism (Bausch + Lomb)	6 (6%)	3741	14.5	8.90	Balafilcon A
PureVision 2 HD for presbyopia (Bausch + Lomb)	2 (2%)	3627	14.0	8.60	Balafilcon A
Air Optix (Alcon)	12 (11%)	3710	14.2	8.60	Lotrafilcon B
Air Optix for astigmatism (Alcon)	4 (4%)	3851	14.5	8.70	Lotrafilcon B
Oasys (Johnson & Johnson)	2 (2%)	3696	14.0	8.40^a^	Senofilcon A
Acuvue Oasys for astigmatism (Johnson & Johnson)	1 (1%)	3880	14.5	8.60	Senofilcon A

^a^ Johnson & Johnson offers the Oasys in two different base curves (BC) and consequently two sagittal depths, but the one used in the study was BC 8.40 mm and CL‐SAG 3696.

### Ocular examination

Participants attended the follow‐up visits having worn the lenses for a minimum of 1 hour. Following visual acuity and over‐refraction measurements, centration, movement in primary gaze and the push‐up recovery test were assessed with a slit lamp (Topcon SL1, topconhealthcare.eu) and recorded using a digital camera (Topcon DC‐1, topconhealthcare.eu). These video recordings were used to assess and grade the following fitting features objectively using proprietary software^14^:

- Horizontal decentration (H‐dec) along the 180° meridian. Positive and negative values correspond to leftward (nasal for the right eye and temporal for the left eye) and rightward (temporal for the right eye and nasal for the left eye) decentration, respectively.
- Vertical decentration (V‐dec) along the 90° meridian. Positive and negative values correspond to superior and inferior decentration, respectively.
- Post‐blink movement in primary gaze (PBM) measured in mm.
- Push‐up recovery speed (PRS) measured in mm/s.

**FIGURE 1:**
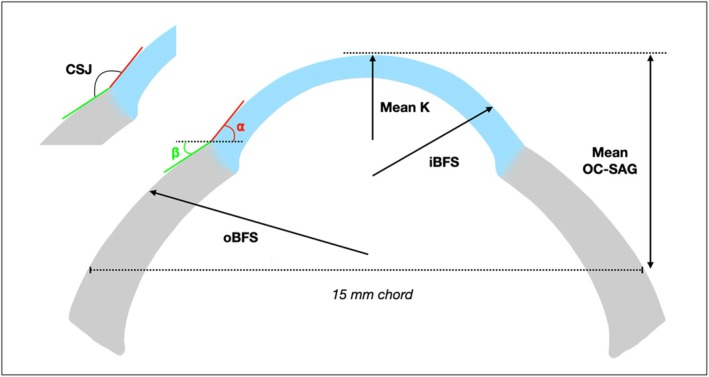
Methodology to calculate the peripheral corneal slope (⍺), the proximal scleral slope (β) and the corneo‐scleral junction (CSJ) angle. Some other ocular parameters are also represented: Mean keratometry (K), inner radius (iBFS), scleral radius (oBFS) and the mean sagittal height of the eye (mean OC‐SAG).

Fluorescein was instilled according to the manufacturer's indications to obtain corneo‐scleral topography using the Eye Surface Profiler (ESP, Eaglet Eye, eaglet‐eye.com).^15^ Three maps were taken and the best one selected in terms of coverage and quality indices to analyse the corneo‐scleral profile. The following parameters were recorded:

- Corneal parameters: Flat and steep keratometry (K), axis of the flat meridian, mean K, eccentricity (e), inner best fit sphere (iBFS—a specific parameter provided by the device, which is a corneal best fit sphere over a 12 mm area), horizontal, vertical and mean visible iris diameter (HVID, VVID and mean VID)
- Corneo‐scleral parameters: Outer radius (oBFS—scleral/outer best fit sphere specifically provided by the ESP), mean 360°, horizontal, vertical, maximum (max) and minimum (min) OC‐SAG for a 15 mm chord (mean OC‐SAG, H‐OC‐SAG, V‐OC‐SAG, max OC‐SAG and min OC‐SAG) (Figure 1), axis of the max and min OC‐SAG meridians, mean 360° OC‐SAG at a chord equal to the lens overall diameter (OAD).

Corneal and scleral parameters obtained with this profilometer have been shown to be reliable and reproducible in a previous publication.^16^

Differences in OC‐SAG values between the mean 360° and the different meridians were calculated, namely the:

- Horizontal and vertical OC‐SAG difference (H‐V OC‐SAG difference)
- Horizontal and vertical OC‐SAG absolute difference (H‐V OC‐SAG abs)
- Horizontal and mean OC‐SAG differences (H‐mean OC‐SAG)
- Horizontal and mean OC‐SAG absolute differences (H‐mean OC‐SAG abs)
- Max–min OC‐SAG difference (max–min OC‐SAG).

The point corresponding to a specific amount of change in curvature between the cornea and the sclera was delimitated over 360° using a design‐purposed algorithm.^10^, ^17^ The slope at the peripheral cornea (α), the anterior sclera (β) and the CSJ angle in the four main quadrants were calculated objectively and automatically using Matlab (MathWorks, mathworks.com), using an algorithm validated in a recent publication^11^ (Figure 1). The following parameters were also recorded: nasal, inferior, temporal, superior and mean 360° peripheral corneal slope (N‐⍺, I‐⍺, T‐⍺, S‐⍺ and mean‐⍺); nasal, inferior, temporal, superior and mean 360° anterior scleral slope (N‐β, I‐β, T‐β, S‐β and mean‐β); nasal, inferior, temporal, superior and mean 360° CSJ angle (N‐CSJ, I‐CSJ, T‐CSJ, S‐CSJ and mean CSJ).

The following differences were also calculated for each eye: temporal versus nasal CSJ, inferior versus superior CSJ, and the ⍺ and β angles: T‐N ⍺, I‐S ⍺, T‐N β, I‐S β, T‐N CSJ and I‐S CSJ.

Lastly, the δ‐sag parameter was calculated as the difference between the CL‐SAG and OC‐SAG at a chord equal to the lens OAD.

### Statistical analysis

Statistical analysis was performed using IBM SPSS software version 29.0.20 (ibm.com). The Kolmogorov–Smirnov test was used to check for normality of the data. Comparison between the right and left eye parameters and lens fitting features was performed using a paired *t*‐Student test as the data were normally distributed.

To ensure greater rigour in the interpretation of statistical significance, a threshold of *p* < 0.01 was adopted for this study. This more conservative criterion reduces the likelihood of Type I errors. Given the potential implications of the findings and the need for robust evidence, a stricter significance level was deemed appropriate. Using *p* < 0.01 increases confidence that observed effects were not due to random variation.

Correlations between corneo‐scleral parameters and the OC‐SAG were established using Pearson's correlation coefficients if both variables followed a normal distribution, or Spearman's correlation coefficients when any of the variables did not pass the Kolmogorov–Smirnov test. The strength of the correlation can be interpreted as follows: values between 0 and ±0.2 indicate a very weak or negligible correlation; between ±0.2 and ±0.4 suggests a weak or mild correlation; between ±0.4 and ±0.6 represents a moderate correlation; between ±0.6 and ±0.8 indicates a strong correlation and values between ±0.8 and ±1.0 reflect a very strong correlation. Associations between ocular parameters and fitting features were analysed in the same way.

Additionally, as the δ‐sag parameter followed a normal distribution, fitting features (PBM, PRS, H‐dec and V‐dec) were analysed for fittings with δ‐sag below, within and above the standard deviation (SD) using ANOVA and post‐hoc Welch tests.

## RESULTS

**FIGURE 2:**
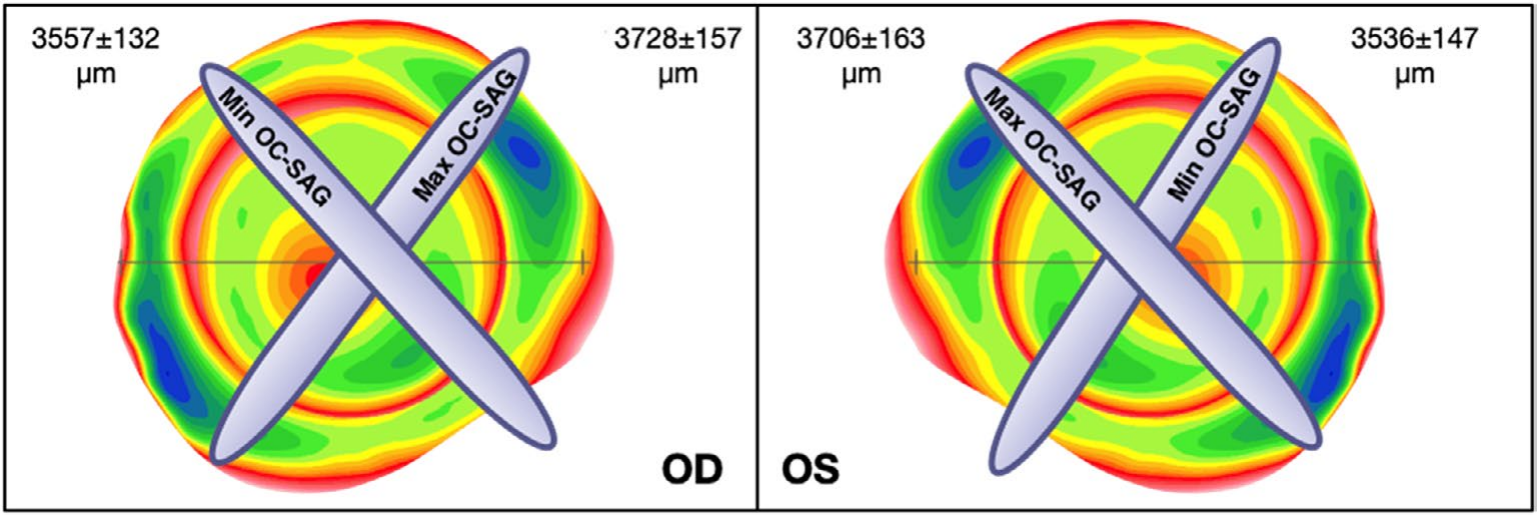
Graphical representation of the maximum and minimum ocular sagittal height (max and min OC‐SAG) values, and their location over 360° for the right and left eyes. Note that the axis of the maximum and minimum values were not located at 90° and were similar in the two eyes after correction for symmetry. OD, right eye; OS, left eye.

Corneal and scleral parameters are described in Table 2. Among the corneal parameters, the flat K, steep K, mean K, iBFS and VVID showed statistically significant differences between the right and left eyes (*p* < 0.01). Mean OC‐SAG at a 15 mm chord diameter was 3638 ± 137 μm (range 3280–3980) and 3617 ± 151 μm (range 3210–4020) for the right and left eyes, respectively. Both values followed a normal distribution and there were no significant differences between the two eyes (*p* > 0.01). In the right eye, the min OC‐SAG was 3557 ± 132 μm and located at 101° ± 51°, while the max OC‐SAG was 3728 ± 157 μm at 62° ± 54°. In the left eye, min and max OC‐SAGs were 3536 ± 147 μm at 72° ± 55° and 3706 ± 163 μm at 97° ± 55°, respectively (Figure 2). Again, the max OC‐SAG and the H OC‐SAG were significantly different between the two eyes (*p* < 0.01), while the remainder of the scleral parameters did not show significant differences between the two eyes (*p* ≥ 0.01).

**TABLE 2:** Corneal and scleral parameters, Kolmogorov–Smirnov (KS) and *t*‐test between both eyes.

	Right eye			Left eye			Normality test passed? α = 0.05	*t*‐Test (*p* value)
	*n*	Mean	KS	*n*	Mean	KS		
Flat K	53	8.04± 0.24 mm (7.45–8.55)	*p* > 0.05	53	8.14 ± 0.25 mm (7.49–8.75)	*p* < 0.05*	RE Yes/LE No	<0.001*
Steep K	53	7.81 ± 0.25 mm (7.23–8.41)	*p* > 0.05	53	7.84 ± 0.28 mm (7.26–8.46)	*p* < 0.05*	RE Yes/LE No	0.003*
Axis	53	87° ± 75° (2–178)	*p* < 0.05*	53	113° ± 76° (0–179)	*p* < 0.05*	No	0.11
Mean K	53	7.92 ± 0.24 mm (7.39–8.48)	*p* > 0.05	53	7.99 ± 0.25 mm (7.40–8.60)	*p* < 0.05*	RE Yes/LE No	<0.001*
e	53	0.49 ± 0.12 (0.24–0.80)	*p* > 0.05	53	0.48 ± 0.08 mm (0.30–0.65)	*p* > 0.05	Yes	0.73
iBFS	53	8.34 ± 0.21 mm (7.80–9.02)	*p* > 0.05	53	8.37 ± 0.22 mm (7.84–9.13)	*p* < 0.05*	RE Yes/LE No	0.005*
HVID	53	12.17 ± 0.44 mm (11.13–13.15)	*p* > 0.05	53	12.27 ± 0.51 mm (11.22–13.93)	*p* < 0.05*	RE Yes /LE No	0.008*
VVID	53	12.29 ± 0.46 mm (11.13–13.18)	*p* > 0.05	53	12.31 ± 0.47 mm (11.22–13.30)	*p* > 0.05	Yes	0.34
Mean VID	53	12.23 ± 0.44 mm (11.13–13.17)	*p* > 0.05	53	12.29 ± 0.48 mm (11.22–13.48)	*p* > 0.05	Yes	0.03
oBFS	53	13.45 ± 0.62 (11.92–14.84)	*p* > 0.05	53	13.33 ± 0.62 (12.27–14.72)	*p* < 0.05*	RE yes/LE No	0.09
Mean OC‐SAG (15 mm)	53	3638 ± 137 (3280–3980)	*p* > 0.05	53	3617 ± 151 (3210–4020)	*p* > 0.05	Yes	0.01
Min OC‐SAG (15 mm)	52	3557 ± 132 (3220–3900)	*p* > 0.05	51	3536 ± 147 (3130–3830)	*p* > 0.05	Yes	0.01
Axis of min OC‐SAG	52	101 ± 51 (1–179)	*p* < 0.05*	51	72 ± 55 (1–177)	*p* < 0.05*	No	0.02
Max OC‐SAG (15 mm)	52	3728 ± 157 (3360–4090)	*p* > 0.05	51	3706 ± 163 (3290–4090)	*p* > 0.05	Yes	0.008*
Axis of max OC‐SAG	52	62 ± 54 (1–176)	*p* < 0.05*	51	97 ± 55 (2–178)	*p* < 0.05*	No	0.004*
H OC‐SAG (15 mm)	52	3655 ± 157 (3330–3990)	*p* < 0.05*	51	3622 ± 165 (3200–4020)	*p* > 0.05	RE No /LE Yes	<0.001*
V OC‐SAG (15 mm)	51	3626 ± 151 (3240–4030)	*p* > 0.05	50	3627 ± 154 (3280–4090)	*p* > 0.05	Yes	0.54
max – min OC‐SAG	52	171 ± 88 (40–450)	*p* > 0.05	51	170 ± 78 (40–430)	*p* < 0.05*	RE Yes/LE No	0.94
H‐V OC‐SAG	51	26 ± 137 (−240–430)	*p* > 0.05	50	−8 ± 131 (−280–400)	*p* > 0.05	Yes	<0.001*
H‐V OC‐SAG abs	51	105 ± 91 (0–430)	*p* < 0.05*	50	96 ± 89 (0–400)	*P* < 0.05*	No	0.44
H‐mean OC‐SAG	52	14 ± 76 (−170–260)	*p* > 0.05	50	7 ± 67 (−170–210)	*p* > 0.05	Yes	0.64
H‐mean OC‐SAG abs	52	57 ± 51 (0–260)	*P* < 0.05*	51	51 ± 43 (0–210)	*p* < 0.05*	No	0.25

*Note*: Corneal parameters: Flat and steep keratometry (K), axis of the flat corneal meridian (axis), Mean K, eccentricity (e), inner best fit sphere (iBFS), horizontal, vertical and mean visible iris diameter (HVID, VVID, mean VID). Scleral parameters: Scleral radius (oBFS), mean minimum, maximum horizontal and vertical sagittal height of the eye (mean OC‐SAG, max OC‐SAG, min OC‐SAG, H OC‐SAG, V OC‐SAG), horizontal and vertical difference (H‐V OC‐SAG), horizontal and vertical absolute difference (H‐V OC‐SAG abs), horizontal and mean difference (H‐mean OC‐SAG), horizontal and mean absolute difference (H‐V OC‐SAG abs).

A single asterisk is referring to *p*‐values representing no normal distribution of data according to the evaluation wih the Kolmogorov‐Smirnov test.

The mean CSJ angles were 177.3° ± 0.7° and 177.5° ± 0.7° in the right and left eyes, respectively. There were statistically significant differences between both eyes (*p* = 0.002). The nasal portion showed the sharpest transition in both eyes with an N‐CSJ of 176.3 ± 1.0 and 176.7° ± 0.8° in the right and left eyes, respectively. These values were significantly different between the two eyes (*p* < 0.001), although this difference was not observed in the rest of the quadrants (*p* ≥ 0.02). Additionally, the T‐N CSJ were significantly different between the two eyes (*p* = 0.009), whereas the I‐S CSJ differences were not significantly different (*p* = 0.52) (Table 3).

**TABLE 3:** Mean, temporal, nasal, superior and inferior corneo‐scleral junction angles (mean CSJ, T‐CSJ, N‐CSJ, S‐CSJ and I‐CSJ); mean, temporal, nasal, superior and inferior peripheral corneal slope (mean‐⍺, T‐⍺, N‐⍺, S‐⍺ and I‐⍺) and mean, temporal, nasal, superior and inferior anterior scleral slope (mean‐β, T‐β, N‐β, S‐β, I‐β). KS, Kolmogorov–Smirnov test. LE, left eye; RE, right eye.

	Right eye			Left eye			Normality test passed? α = 0.05	*t*‐Test (*p* value)
	*n*	Mean (º) (range)	KS	*n*	Mean (º) (range)	KS		
Mean CSJ	53	177.3 ± 0.7 (175.5–178.9)	*p* > 0.05	53	177.5 ± 0.7 (176.1–179.1)	*p* > 0.05	Yes	0.002*
T‐CSJ	53	177.9 ± 1.2 (173.7–180)	*p* < 0.05*	53	177.8 ± 1.1 (174.4–180)	*p* < 0.05*	No	0.81
N‐CSJ	53	176.3 ± 1.0 (173.1–178.1)	*p* > 0.05	53	176.7 ± 0.8 (175.3–178.4)	*p* > 0.05	Yes	<0.001*
S‐CSJ	53	177.8 ± 1.0 (175.5–179.9)	*p* < 0.05*	53	178.1 ± 0.8 (175.8–179.8)	*p* > 0.05	No RE/Yes LE	0.02
I‐CSJ	53	177.7 ± 1.0 (175.0–179.5)	*p* > 0.05	53	177.9 ± 1.0 (173.6–179.9)	*p* < 0.05*	Yes RE/No LE	0.27
T‐N CSJ	53	1.5 ± 1.2 (−2.1–4.7)	*p* > 0.05	53	1.1 ± 1.1 (−2.7–3.1)	*p* > 0.05	Yes	0.009*
I‐S CSJ	53	−0.1 ± 1.3 (−2.5–3.7)	*p* > 0.05	53	1.2 ± 1.0 (−2.5–3.3)	*p* > 0.05	Yes	0.52
Mean‐⍺	53	38.5 ± 1.6 (34.5–42.3)	*p* > 0.05	53	38.4 ± 1.7 (33.7–42.4)	*p* > 0.05	Yes	>0.05
T‐⍺	53	42.7 ± 2.1 (38.7–47.2)	*p* > 0.05	53	42.7 ± 2.1 (36.8–47.1)	*p* > 0.05	Yes	0.80
N‐⍺	53	35.5 ± 2.3 (29.8–39.3)	*p* < 0.05*	53	34.8 ± 2.6 (28.3–39.7)	*p* > 0.05	RE No/LE Yes	<0.001*
S‐⍺	53	36.0 ± 2.3 (31.6–40.7)	*p* > 0.05	53	36.2 ± 2.7 (31.9–46.7)	*p* > 0.05	Yes	0.37
I‐⍺	53	39.7 ± 2.1 (35.6–43.7)	*p* > 0.05	53	39.9 ± 2.0 (35.3–43.2)	*p* > 0.05	Yes	0.46
T‐N ⍺	53	7.2 ± 2.7 (1.4–13.2)	*p* > 0.05	53	7.9 ± 3.1 (−0.2–14.4)	*p* > 0.05	Yes	0.05
I‐S ⍺	53	3.8 ± 2.5 (−5.0–10.4)	*p* > 0.05	53	3.7 ± 2.8 (−7.5–9.1)	*p* > 0.05	Yes	0.87
Mean‐β	53	35.9 ± 1.8 (31.7–40.3)	*p* > 0.05	53	36.0 ± 1.9 (31.2–40.2)	*p* > 0.05	Yes	0.78
T‐β	53	40.4 ± 2.4 (36.0–46.1)	*P* > 0.05	53	40.5 ± 2.6 (34.2–47.1)	*p* > 0.05	Yes	0.83
N‐β	53	31.8 ± 2.7 (23.8–36.4)	*P* > 0.05	53	31.4 ± 2.8 (23.5–36.1)	*p* > 0.05	Yes	0.09
S‐β	53	33.9 ± 2.3 (29.7–39.2)	*p* > 0.05	53	34.4 ± 2.8 (30.1–41.8)	*p* > 0.05	Yes	0.04
I‐β	53	37.6 ± 2.3 (31.9–41.7)	*P* > 0.05	53	37.9 ± 2.3 (31.1–41.8)	*p* > 0.05	Yes	0.23
T‐N β	53	8.6 ± 3.0 (0.9–14.5)	*p* > 0.05	53	9.1 ± 3.3 (2.2–17.9)	*p* > 0.05	Yes	0.23
I‐S β	53	3.7 ± 2.4 (−2.3–8.7)	*p* > 0.05	53	3.5 ± 3.3 (−6.8–10.0)	*p* > 0.05	Yes	0.65

*Note*: Temporal/nasal and inferior/superior differences are also calculated in this table.

A single asterisk is referring to *p*‐values representing no normal distribution of data according to the evaluation wih the Kolmogorov‐Smirnov test.

The CSJ angle was calculated on the basis of the α and β angles. Table 3 shows the mean angles and the values divided by quadrants. As expected, the peripheral cornea was steeper with a mean‐⍺ around 38°, while the proximal sclera showed a flatter mean‐β of about 36° in both eyes. Interestingly, and following the pattern of the CSJ angle, there were also statistically significant differences between the N‐⍺ of the two eyes (*p* < 0.001), while the remainder of the quadrants were not significantly different (*p* ≥ 0.37). When the scleral slope was analysed by quadrants, there were no significant differences between the two eyes (*p* ≥ 0.04).

Table 4 shows correlations between some of the corneal and corneo‐scleral parameters and the OC‐SAG. Parameters presumed to have a greater impact on OC‐SAG were selected to test for associations. Among these were central corneal curvature (mean K), overall corneal curvature (iBFS), eccentricity (e), mean VID, mean CSJ and the mean ⍺ and β angles. Curvature parameters such as the mean K and the iBFS revealed a moderate and strong inverse correlation, respectively, with the OC‐SAG in both eyes. Mean‐⍺ and β angles also showed a very strong direct correlation with both parameters. Eccentricity was inversely and strongly correlated in the right eye (*r* = −0.52, *p* < 0.001) but not in the left eye (*r* = −0.12, *p* = 0.40). The mean VID showed no significant correlation in the right eye (*r* = 0.18, *p* = 0.19) and a mild direct correlation in the left eye only (*r* = 0.29, *p* = 0.04). Finally, no significant correlation was found in either eye between the mean CSJ and the OC‐SAG (*p* ≥ 0.56).

**TABLE 4:** Correlations between some corneo‐scleral parameters and the mean sagittal height of the eye (mean OC‐SAG) on each eye.

	Right eye	Left eye
Mean K	*r* = −0.421 (*p* = 0.002)*	ρ = −0.353 (*p* = 0.01)*
e	*r* = −0.519 (*p* < 0.001)**	*r* = −0.117 (*p* = 0.40)
iBFS	*r* = −0.782 (*p* < 0.001)**	ρ = −0.641 (*p* < 0.001)**
Mean VID	*r* = 0.182 (*p* = 0.19)	*r* = 0.290 (*p* = 0.04)
Mean CSJ	*r* = −0.083 (*p* = 0.56)	*r* = 0.050 (*p* = 0.73)
Mean ⍺	*r* = 0.838 (*p* < 0.001)**	*r* = 0.889 (*p* < 0.001)**
Mean‐β	*r* = 0.791 (*p* < 0.001)**	*r* = 0.839 (*p* < 0.001)**

*Note*: Mean keratometry (K), eccentricity (e), inner best fit sphere (iBFS), mean visible iris diameter (mean VID), mean corneo‐scleral junction (CSJ) angle, mean peripheral corneal slope (⍺) and mean anterior scleral slope (β). Moderate correlations are marked with (*) and strong and very strong correlations with (**).

The fitted contact lens parameters were those recommended by the manufacturer for each of the standard lenses. Thus, the diameter was between 14 and 14.5 mm (14–14.2 mm for spherical and multifocal lenses and 14.5 mm for toric lenses) and the base curve ranged from 8.4 to 8.9 mm, without any significant differences between the two eyes. The spherical equivalent was −2.04 ± 2.70 D in the right eye and −1.83 ± 2.91 D in the left eye. The CL‐SAG was also similar in both eyes, with a mean value of 3774 ± 88 and 3767 ± 88 μm in the right and left eyes, respectively (Table 5). None of the contact lens parameters were significantly different between the two eyes (*p* ≥ 0.05). When the lens fitting features were analysed, δ‐sags of 411 ± 122 and 427 ± 136 μm were found in the right and left eyes, respectively, with a normal distribution (*p* < 0.05 for both eyes) and without significant differences between the two eyes (*p* ≥ 0.05). In the same way, there were no significant differences in the lens fitting features between the two eyes (*p* ≥ 0.05). There were no statistically significant correlations between the δ‐sag and the lens fitting features in either eye (*p* ≥ 0.35). Some mild and moderate associations between the lens fit and ocular parameters were found and are summarised in Table 6.

**TABLE 5:** Contact lens (CL) parameters: overall lens diameter (OAD), base curve (BC), spherical equivalent, sagittal depth of the lenses (CL‐SAG) and sagittal height of the eye at a chord equal to the OAD (OC‐SAG at lens OAD). KS, Kolmogorov–Smirnov test; LE, left eye; RE, right eye.

	Right eye			Left eye			Normality test passed? α = 0.05	t‐test (*p* value)
	*n*	Mean (range)	KS	*n*	Mean (range)	KS		
OAD	53	14.26 ± 0.22 mm (14–14.50)	*P* < 0.05*	53	14.24 ± 0.22 mm (14–14.50)	*P* < 0.05*	No	0.49
BC	53	8.63 ± 0.10 mm (8.40–8.90)	*p* < 0.05*	53	8.63 ± 0.10 mm (8.40–8.90)	*p* < 0.05*	No	1.00
Spherical equivalent	53	−2.04 ± 2.70 D (−8.00–6.63)	*P* < 0.05*	53	−1.83 ± 2.91 D (−9.00–6.38)	*p* < 0.05*	No	0.15
CL‐SAG	53	3774 ± 88 μm (3627–3969)	*P* < 0.05*	53	3767 ± 88 μm (3627–3969)	*P* < 0.05*	No	0.44
OC‐SAG at lens OAD	53	3363 ± 124 μm (3130–3660)	*p* > 0.05	53	3340 ± 140 μm (3040–3660)	*p* > 0.05	Yes	0.05
δ‐sag	53	411 ± 122 μm (128–734)	*p* > 0.05	53	427 ± 136 μm (88–814)	*p* > 0.05	Yes	0.05
PBM	53	0.87 ± 0.48 mm (0.19–1.94)	*P* < 0.05*	52	0.92 ± 0.58 mm (0.13–2.08)	*p* > 0.05	No RE/Yes LE	0.64
PRS	53	1.82 ± 1.13 mm/seg (0.17–4.83)	*p* > 0.05	49	1.48 ± 1.12 mm/seg (0.17–5.69)	*P* < 0.05*	Yes RE/No LE	0.24
H‐dec	53	−0.02 ± 0.13 mm (−0.25 to 0.29)	*p* > 0.05	53	−0.08 ± 0.15 mm (−0.38 to 0.25)	*p* > 0.05	Yes	0.04*
V‐dec	53	0.01 ± 0.16 mm (−0.34–0.46)	*p* > 0.05	53	0.00 ± 0.17 mm (−0.54–0.34)	*p* > 0.05	Yes	0.72

*Note*: Lens fitting features: δ‐sag (difference between the CL‐SAG and OC‐SAG) , post‐blink movement (PBM), push‐up recovery speed (PRS), horizontal decentration (H‐dec) and vertical decentration (V‐dec). The δ‐sag was calculated using the OC‐SAG at a chord equal to the lens OAD.

A single asterisk is referring to *p*‐values representing no normal distribution of data according to the evaluation wih the Kolmogorov‐Smirnov test.

**TABLE 6:** Correlations between lens fitting features and ocular parameters.

Lens fitting variable	Ocular/CL parameter	Correlation coefficient	Significance
PBM in right eye	H OC‐SAG	ρ = −0.273	*p* = 0.05
	I‐⍺	ρ = −0.283	*p* = 0.04
	I‐S ⍺	ρ = −0.279	*p* = 0.04
	I‐β	ρ = −0.339*	*p* = 0.01
	I‐S β	ρ = −0.430*	*p* = 0.001
PRS in right eye	N‐CSJ	*r* = 0.291	*p* = 0.04
	I‐β	*r* = −0.294	*p* = 0.03
	I‐S β	*r* = −0.287	*p* = 0.04
V‐dec in left eye	VVID	*r* = 0.413*	*p* = 0.002
	Mean VID	*r* = 0.339*	*p* = 0.01
	H‐V OC‐SAG	*r* = −0.316*	*p* = 0.03
	H‐mean OC‐SAG	*r* = 0.299	*p* = 0.04
PBM in left eye	oBFS	ρ = −0.365*	*p* = 0.008
	Mean CSJ	*r* = 0.322*	*p* = 0.02
	S‐CSJ	*r* = 0.334*	*p* = 0.02
	I‐CSJ	ρ = 0.316*	*p* = 0.02
PRS in left eye	oBFS	ρ = −0.449*	*p* = 0.001

*Note*: Moderate correlations are marked with (*). No strong or very strong correlations were found. Post‐blink movement (PBM), push‐up recovery speed (PRS), vertical decentration (V‐dec), horizontal sagittal height of the eye (H OC‐SAG), inferior peripheral corneal slope (I‐⍺), inferior/superior difference in peripheral corneal slope (I‐S ⍺), inferior anterior scleral slope (I‐β), inferior/superior difference in anterior scleral slope (I‐S β), nasal corneoscleral junction angle (N‐CSJ), vertical visible iris diameter (VVID), mean visible iris diameter (mean VID), horizontal/vertical difference in sagittal height of the eye (H‐V OC‐SAG), horizontal/mean difference in sagittal height of the eye (H‐mean OC‐SAG), scleral radius (oBFS), mean corneoscleral junction angle (mean CSJ), superior corneoscleral junction angle (S‐CSJ), inferior corneoscleral junction.

**FIGURE 3:**
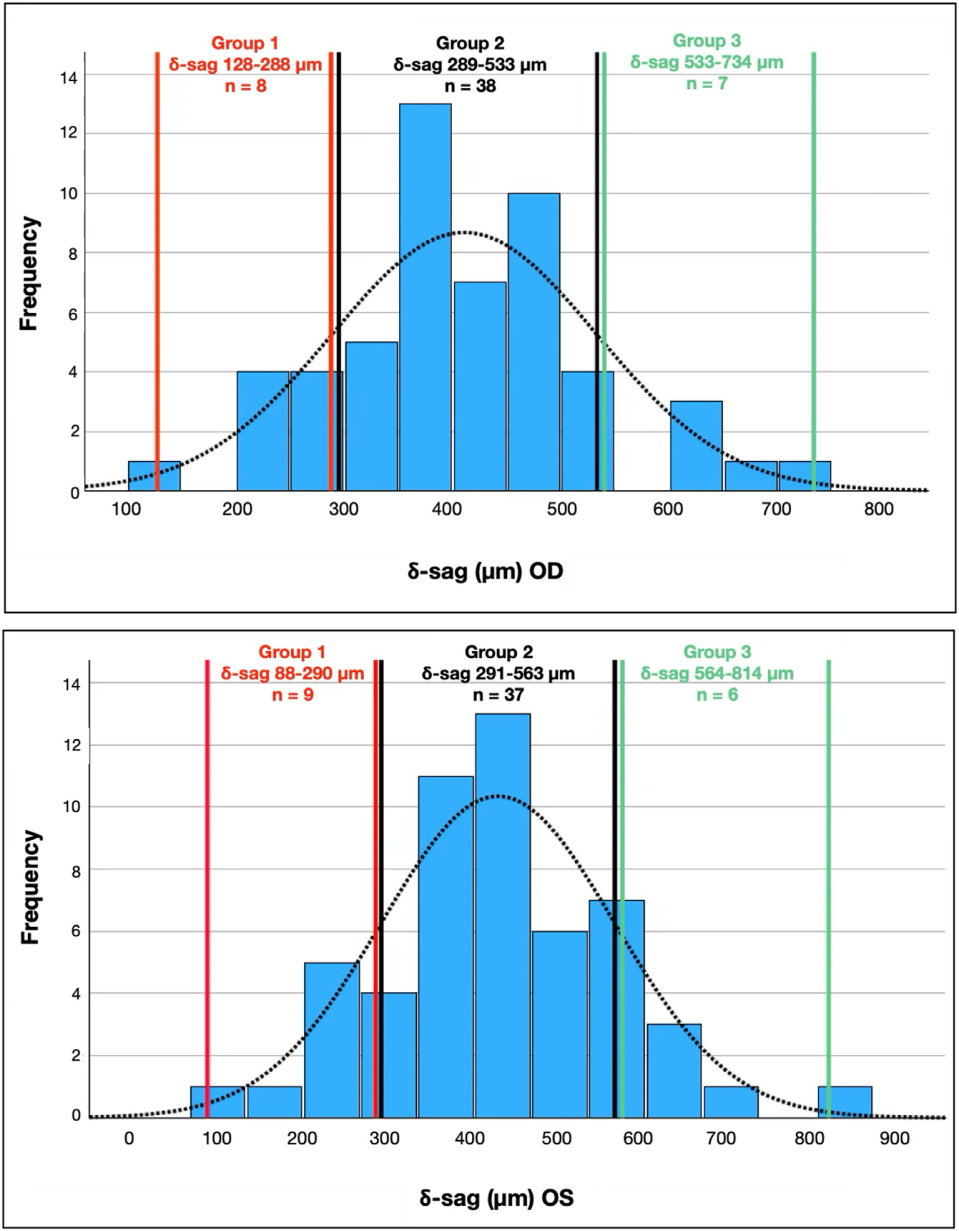
Graphs showing the normal distribution of 𝛿‐sag for the right eye (OD, top figure) and left eye (OS, bottom figure), and the distribution of the three groups of 𝛿‐sags on each eye (i.e., below, within and greater than the standard deviation).

Lens fitting features were analysed separately for three groups of δ‐sags. The first group included fittings with δ‐sags below the SD (Group 1), the second group included those with δ‐sags within the SD (Group 2) and the third group included fittings with δ‐sag above the SD (Group 3) (Figure 3). As shown in Table 7, the analysis did not reveal significant differences between the lens fitting features in the three groups.

**TABLE 7:** Lens fitting features, post‐blink movement (PBM), push‐up recovery speed (PRS), horizontal decentration (H‐dec) and vertical decentration (V‐dec) analysed separately for the three groups of 𝛿‐sag (difference between the CL‐SAG and the OC‐SAG).

	Right eye				Left eye			
	𝛿‐sag < mean‐SD (*n* = 8)	𝛿‐sag = mean ± SD (*n* = 38)	𝛿‐sag > mean + SD (*n* = 7)	ANOVA/Welch	𝛿‐sag < mean‐SD (*n* = 9)	𝛿‐sag = mean ± SD (*n* = 38)	𝛿‐sag > mean + SD (*n* = 6)	ANOVA/Welch
𝛿‐sag (μm)	228 ± 49	411 ± 63	622 ± 71	<0.001*	228 ± 61	437 ± 73	667 ± 83	<0.001*
PBM (mm)	0.66 ± 0.23	0.88 ± 0.49	1.05 ± 0.57	0.11	0.67 ± 0.49	0.98 ± 0.59	0.90 ± 0.57	0.35
PRS (mm/s)	1.55 ± 1.20	1.89 ± 1.07	1.77 ± 1.52	0.74	0.97 ± 0.49	1.60 ± 1.25	1.59 ± 0.96	0.32
H‐dec (mm)	−0.04 ± 0.12	−0.04 ± 0.13	0.10 ± 0.12	0.03	−0.07 ± 0.14	−0.09 ± 0.15	−0.04 ± 0.15	0.76
V‐dec (mm)	−0.03 ± 0.10	0.01 ± 0.14	0.07 ± 0.28	0.60	0.01 ± 0.21	−0.01 ± 0.18	0.04 ± 0.06	0.81

*Note*: There were no differences between the three groups.

The single asterisk is representing *p*‐values showing statistical significance.

**FIGURE 4:**
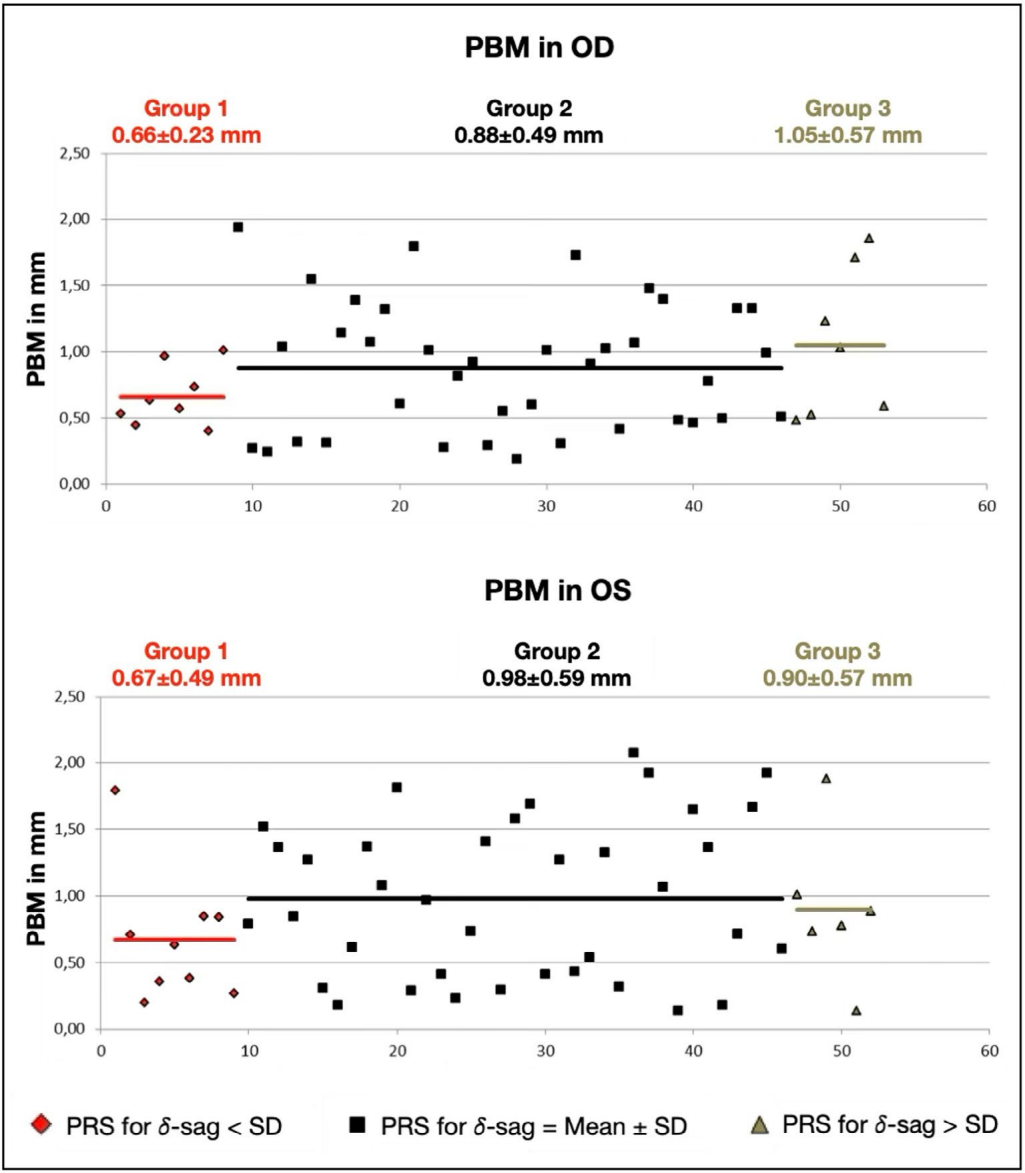
Graphical representation of post‐blink movement (PBM) in the right and left eyes for different delta‐sag values. PBM values with 𝛿‐sag below the standard deviation (SD) are shown in red. PBM values for fittings with 𝛿‐sag in the mean ± SD range are shown in black. PBM in fittings with 𝛿‐sag greater than the SD are shown in green. The red, black and green lines represent the mean PBM value for the respective groups. OD, right eye; OS, left eye.

**FIGURE 5:**
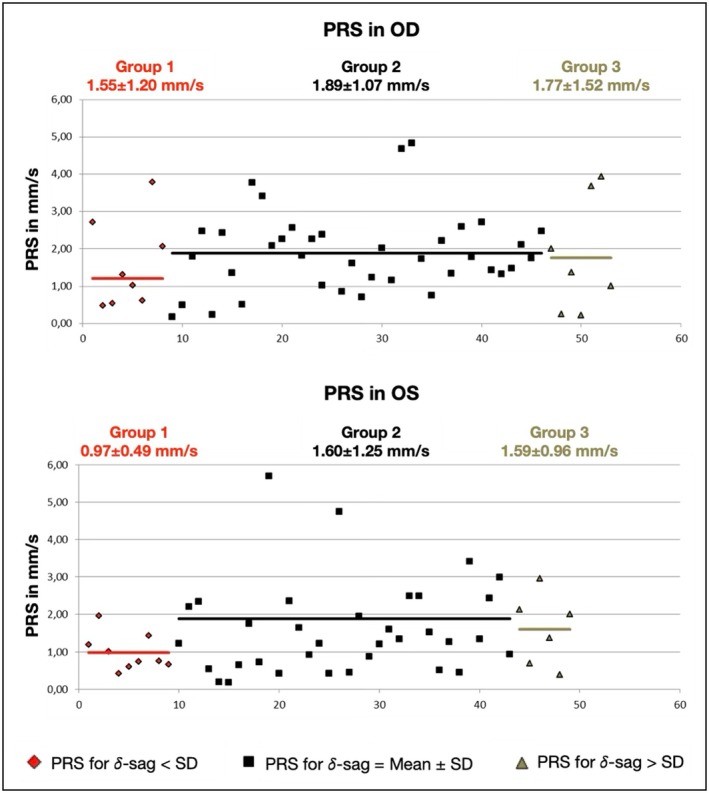
Graphical representation of push‐up recovery speed (PRS) in the right and left eyes for different 𝛿‐sag values. Red, black and green represent values below, within and above the mean ± SD range, respectively. The red, black and green lines represent the mean PRS values for the respective groups. OD, right eye; OS, left eye.

Figures 4 and 5 show a graphical representation of the PBM and the PRS for each lens fitting, and the mean values for each group. Although there were no significant differences in these lens fitting features among the three groups, the mean values showed some consistent differences. PBM was consistently lower in Group 1 (lower δ‐sag) than Group 2 (mean δ‐sag), being 0.22 and 0.31 mm in the right and left eyes, respectively. Differences in PBM between Groups 2 and 3 (higher δ‐sag) were lower and not consistent between the two eyes. In terms of the PRS, Group 1 again showed consistently lower mean values than Group 2 in both eyes, specifically 0.34 and 0.63 mm/s lower in the right and left eyes, respectively. Group 3 also showed less movement in both eyes, but the difference was minimal (0.12 and 0.01 mm/s in the right and left eyes, respectively).

## DISCUSSION

### Ocular surface shape

The first part of this study defined the corneo‐scleral profile in a group of standard soft contact lens wearers. Corneal parameters have been widely defined in previous studies^5^, ^7^ and some differences were observed that can be attributed to the use of different devices and techniques of measurement.^5^, ^7^ However, the corneal values obtained in the present study are similar to those obtained with the ESP in a previous report.^18^ Interestingly, there were statistically significant differences between the two eyes for curvature parameters (flat K, steep K, mean K and iBFS) and in the HVID (Table 2). Although these differences have not been studied in detail, they have been considered in previous reports on the on‐eye soft contact lens features, with little or no clinical significance being found.^5^

Regarding the scleral parameters, the mean OC‐SAG for a 15 mm chord diameter was ~3600 μm, with no statistically significant differences being observed between the two eyes (Table 2). Recorded ESP values were in the same range as previous reports,^12^, ^18^ for example, Ritzmann et al. obtained findings with the AS‐OCT of ~3700 μm^7^, ^19^, ^20^, ^21^ and Kern and Bataille found values close to ~3800 μm using the Pentacam CSP.^22^, ^23^ Such differences using different instruments have also been documented previously.^24^ There were also differences between the OC‐SAG values in the horizontal/vertical meridians and the mean value, which is in line with previous reports showing differences of <100 microns in different meridians.^19^, ^25^ The difference between the max and min OC‐SAG was approximately 170 microns and the axis of the min OC‐SAG was located close to 100° and 70° in the right and left eyes, respectively (Table 2). In that regard, Visser et al. found the axis of stabilisation for the flattest meridian of scleral lenses at 137° and 47° in the right and left eyes, respectively.^26^ Although these indicate differences from the findings of the current study (Visser et al.^26^ used scleral lenses, presumably fitted in pathological eyes), the ‘flat’ axis does not align with the horizontal or vertical meridian, which confirms an oblique tendency in scleral asymmetry.

Therefore, fitting soft lenses based on a single horizontal meridian would not be as accurate as using the mean value obtained over 360°. Other relevant findings observed here were that the flattest and steepest meridians were not located 90° apart and the axis of the main meridians was similar in both eyes after correction for symmetry.

When the corneo‐scleral transition was analysed, as might be expected, the peripheral cornea was steeper than the proximal sclera in both eyes. Mean CSJ values were very similar to those obtained in previous studies.^7^, ^11^ Using AS‐OCT, the nasal portion also showed the most abrupt transition in both eyes.^7^ However, perhaps the most relevant finding was statistically significant differences in the mean CSJ between the two eyes (*p* = 0.002), with a transition that was slightly sharper in the right eye. When the CSJ was analysed by quadrants, the nasal portion was significantly sharper in the right eye by about 0.5° (*p* < 0.001), but this difference was not observed in the remaining quadrants (*p* ≥ 0.02). The difference seems to come from the nasal peripheral corneal slope (nasal‐⍺), which was steeper in the right eye (35.5° ± 2.3°) than the left eye (34.8° ± 2.6°) (*p* < 0.001). In contrast, there were no significant differences between the two eyes for the anterior scleral slope (β), neither in the mean values nor by quadrants (*p* ≥ 0.04) (Table 3). While these differences have not been described previously, they could be explained by the high variability in the insertion of the rectus muscles. While the medial rectus insertion is the closest to the limbus,^27^ observations have shown either large inter‐individual variations^28^, ^29^, ^30^, ^31^ or differences between the same rectus muscle in the two eyes of an individual.^32^ These differences between the right and left eyes have been mentioned previously, but have not been discussed thoroughly. An additional fact that supports this finding is that a former scleral lens manufacturer offered a trial set with different lenses for the right and left eye for several years. After many scleral lens fittings, the laboratory found differences in the anatomical corneo‐scleral shape of the two eyes and manufactured a data‐driven diagnostic set with different designs for the two eyes.^33^ Importantly, scleral lenses are mainly used for irregular and pathological eyes, while the present study was conducted on healthy eyes. Nevertheless, this observation may support the current findings and have clinical implications for scleral lens fittings. However, any clinical impact for soft lens fitting remains to be determined.

### Correlation of the soft lens fit with the ocular surface profile

Secondly, the absence of a significant correlation with central corneal parameters was noted, while some correlations with the peripheral corneo‐scleral transition were determined.

As stated previously, it has been proposed that soft lenses be fitted based on the sagittal height and δ‐sag.^8^ However, there is no consensus as to which chord should be used to obtain OC‐SAG. Here, CL‐SAG values were measured for the OAD of each lens.^13^ In fact, if all CL‐SAG values were recalculated for a 14 mm chord diameter, then the differences between lenses would decrease.^34^ Two additional factors which would impact CL‐SAG are edge strain and temperature. For instance, soft lenses flex on the eye and an edge strain of 3% is expected, which would increase the lens diameter from 14.0 to 14.4 mm.^8^, ^35^ However, when the lenses are measured at on‐eye temperature, they shrink, showing a decrease in OAD and consequently a decrease in CL‐SAG.^36^ Most daily disposable soft lenses showed a decrease in OAD > 0.2 mm when they were measured at 34°C, compared with the same measure at room temperature (20°C).^36^ We considered that the assumption of an increase in OAD due to lens flexure^35^ could be compensated by tangible evidence of an OAD decrease at on‐eye temperature.^36^ Then, δ‐sag was calculated for OC‐SAG at a chord diameter equal to the OAD of the lens worn by each participant. Some information regarding the optimal δ‐sag for custom soft lenses has been provided by Montani et al. and Michaud et al.^37^ However, there is little information as to what would be the optimal δ‐sag for standard lenses. This study described the δ‐sag in a group of standard reusable (monthly or bi‐weekly) mid‐ to long‐term soft contact lens users, with a goal of ~400 μm, a normal distribution and most fits between 300 and 550 μm. Nevertheless, as reported above, OC‐SAG may vary depending on the device being used to measure this parameter.^7^, ^12^, ^18^, ^19^, ^20^, ^21^, ^22^, ^23^, ^38^ Consequently, the target might drop to the ~200–300 μm range when the OC‐SAG is measured with devices other than the ESP. When analysing the relationship with δ‐sag, it should be noted that lenses were not randomly assigned, but rather selected by an eye care professional based on the ocular and refractive characteristics of the individual eye. This may represent a source of bias, as some lenses—particularly toric designs, which typically have a diameter close to 14.5 mm—also tend to exhibit a higher CL‐SAG. However, this potential bias was minimised by measuring the OC‐SAG at a chord corresponding to the overall diameter (OAD) of each lens. Nonetheless, the results may still be influenced by the prescribing tendencies of the clinician who originally fitted the lenses, as they could also influence the lens fitting features as discussed below.

The on‐eye lens fitting features were analysed objectively with proprietary software and the results in terms of H and V‐dec were similar to those obtained subjectively by an experienced examiner in previous reports.^7^ The PBM was 0.5 mm higher than subjective values and significantly above the range considered optimum in previous reports (0.2–0.6 mm)^3^, ^7^ (Table 5). This difference may be explained by some calibration issues that were detected because the HVID values provided by the software were smaller than the values obtained with the ESP. The videos were analysed retrospectively. Further, the magnification used in the recording might not match the software settings perfectly. Nevertheless, it should be kept in mind that the deviation in PBM can also be attributed to the fact that it was measured objectively and compared with subjective findings. PRS values were expressed in mm/s, while most previous reports graded the ease of push‐up on a subjective scale from 0 to 100,^3^, ^7^ which has been found to contribute less to overall lens fit.^39^ Perhaps the most significant finding was that the lens fitting feature variables were similar in the two eyes, except for H‐dec which was minimally temporal in the right eye (−0.02 ± 0.13 mm) and more pronounced nasally in the left eye (−0.08 ± 0.15 mm) (Table 5). When this is analysed in conjunction with the previous description of statistically significant differences in the H OC‐SAG and the T‐N CSJ profile of each eye, it increases the clinical relevance of the findings.

Associations between the lens fitting features and ocular parameters were analysed, and a lack of consistency between the correlations found in the two eyes was noted. However, a common pattern was that the dynamic features of the lens fitting (PBM and PRS) showed significant correlations with the corneo‐scleral transition rather than with central corneal parameters or the VID. This is in line with previous reports,^5^, ^6^, ^7^ which suggested that peripheral ocular parameters may have more influence on the fit than the traditionally used central parameters. This makes sense based on the present findings showing that the peripheral parameters also had a greater influence on the mean OC‐SAG (Table 4). Interestingly, the PBM, which is vertical, was correlated with the inferior and superior corneo‐scleral profile in both eyes. In the right eye, it was inversely correlated with the inferior ⍺ and β angles and the difference between these angles in the inferior and superior quadrant, while in the left eye, there were direct associations with the CSJ angle. This would mean that the lens moves more in eyes having either a less pronounced slope at the inferior cornea and sclera or a smaller difference between the slope in the inferior and superior quadrants, based on the right eye findings. If one considers the left eye findings, a more linear or smooth transition between the cornea and the sclera in the mean‐360° values, and specifically in the inferior/superior quadrants, would result in greater lens movement. The PRS was also inversely correlated with the scleral profile in the right eye in a similar manner to the PBM. Although not fully congruent, it was also inversely correlated with the overall scleral radius (oBFS) in the left eye. Significant correlations with H‐dec were not found in either eye, while V‐dec was moderately and directly correlated with VVID and H‐V OC‐SAG. An interpretation could be that for a larger VVID, a more superior or less inferior decentration can be expected, and larger differences between H and V OC‐SAG might result in less superior or more inferiorly decentred lenses (Table 6).

No significant associations were found between δ‐sag and the lens fit in the current participants. It is important to note that the subjects included here were successful standard soft lens wearers. Although corneal and scleral parameters showed a wide range of values, they were presumably normal eyes in terms of the soft lens fitting. Therefore, to establish a fit pattern for different δ‐sags values, the lens fitting variables were analysed into three groups having different δ‐sags. This analysis should be interpreted with caution due to several limitations. First, the Group 2 findings do not indicate that the fitting was more optimal in terms of lens fitting characteristics or comfort. In fact, a standardised assessment of comfort was not available in this study. Group 2 simply represents the central range of δ‐sag, and the goal was to explore potential fitting patterns both within and outside this range. Second, as previously mentioned, the number of fittings outside the central range (Groups 1 and 3) was low and should be considered a major limitation of this analysis. The general pattern seems to be that the lens moves less (in terms of mean values) in the group with lower δ‐sags (about 0.20–0.30 mm in PBM and 0.30–0.50 mm/s in PRS). It also appears that the lenses tend to move less in the group with higher δ‐sags, although the differences were much lower (<0.12 mm). Indeed, the PBM in Group 3 of the right eye was higher. While these differences between mean values could be clinically significant, statistical analysis did not indicate differences between the three groups except for H‐dec in Groups 2 and 3 of the right eyes. Based on this analysis, the lenses tended to decentre more temporally in the group with higher δ‐sags, but only for the right eye data (Table 7 and Figures 3, 4, 5).

Although some studies have reported associations between lens fit and δ‐sag,^40^ others have not identified such a relationship.^41^ In the present investigation, participants were fitted with different types of lenses, which complicates the ability to isolate the influence of individual lens parameters. The CL‐SAG may represent the parameter that most comprehensively characterises the overall shape of a soft contact lens; however, it does not account for the design of the lens periphery. The present findings indicate that peripheral corneo‐scleral parameters were associated with lens fit, suggesting that variations in lens peripheral design may have an impact on the fitting outcomes. Consequently, when assessing the potential associations cited here between δ‐sag and lens fit, the use of multiple lens types should be acknowledged as a potential limitation. While standard soft lenses have different δ‐sag values, they are also manufactured using different materials and designs. Therefore, this limitation should not be considered specific to this study, but rather an inherent shortcoming when attempting to establish associations between standard soft contact lenses and lens fitting.

In addition, age‐related changes in the tear film of presbyopic participants, along with the heterogeneity of the sample, can impact lens fitting and should also be considered as a limitation of this study.

## CONCLUSION

The on‐eye soft lens fit continues to be a complicated issue to predict. This study supports the greater influence of peripheral corneo‐scleral parameters on the soft lens fit and the absence of an association with central corneal parameters. A description of some patterns of soft lens fitting features related to corneo‐scleral parameters has been provided, but they were not consistent between the two eyes. This lack of consistency could be attributed to the complexity of the on‐eye soft lens performance. Other variables, such as lid tension, lens flexure and/or the peripheral design of the lenses were not considered here due to the retrospective nature of the study. Nevertheless, in the light of these results, future studies should focus on peripheral parameters of the eye and lens fit. Lens movement in terms of PBM and PRS seems to be influenced to a greater extent by the corneo‐scleral transition at the superior and inferior quadrants. Therefore, it would make sense to focus on the slopes in these areas, rather than looking at central corneal parameters or even the sagittal height.

## AUTHOR CONTRIBUTIONS

**Javier Rojas Viñuela:** Conceptualization; data curation; formal analysis; investigation; methodology; writing – original draft. **James S. Wolffsohn:** Software; resources; writing – review and editing. **Alejandra Consejo:** Software; resources; writing – review and editing. **Eef van der Worp:** Resources; writing – review and editing. **David P. Piñero:** Supervision; visualization; writing – review and editing.

## CONFLICT OF INTEREST STATEMENT

The authors have no financial or proprietary interests in any materials or methods discussed, and they declare no financial disclosures.

## FUNDING INFORMATION

No author has a financial or proprietary interest in any of the materials or methods mentioned. The authors have no financial disclosures to declare.
